# A comprehensive review of DC microgrid in market segments and control technique

**DOI:** 10.1016/j.heliyon.2022.e11694

**Published:** 2022-11-17

**Authors:** A. Ashok Kumar, N. Amutha Prabha

**Affiliations:** School of Electrical Engineering, Vellore Institute of Technology, Vellore, India

**Keywords:** Microgrid, Market segment, Hierarchical control, Primary control, Secondary control, Territory control

## Abstract

DC- Microgrid has been widely developed for the distribution system. Energy utilizing device is easily integrated on DC – Microgrid to minimize losses in ease. In recent years, due to power distribution, -multiple energy sources are connected to DC-Microgrid. The connection of multi-sources deviates in power sharing and voltage performance is the significant challenges. To enhance the good quality of power and higher system efficiency, control strategies play a vital role. The main focus of this work is on DC-Microgrid control techniques. The conventional droop control has low current sharing and accuracy. To sort out the drawbacks, an improved primary controller is enhanced for better power sharing and accuracy. To achieve stable and dynamic current sharing scheme, secondary control strategies are reviewed. Further Multi-layer and Virtual control strategies for good voltage regulation have been reviewed. The purpose of this review is to represent on the hierarchical control structure of the DC microgrid and its three-level control architecture and this study explores distributed, centralized, decentralized, and hierarchical control techniques and discusses their strengths and weakness.

## 1 Introduction

The power converter interfaced with distributed energy resources includes wind generator [1], photo voltaic [2], energy storage systems [3], and micro turbine system [4]. It also provides the basis for self-sustaining entities called microgrid. Consequently, the idea of the microgrid (MG) was developed at the start of the 20th century in order to integrate the fundamental difference between the traditional grid and distributed generation (DG) units, thereby taking control of the distributed electricity source. Microgrids are classified as DC-Microgrid or AC-Microgrid [5].

**Table 1:** Comparison of Decentralized, Centralized and distribution control.

Features	Decentralized [50, 51]	Centralized [54, 55, 56]	Distribution [52, 53]
Reliability	High	Low	Medium
Modularity	High	Low	High
Communication	Power control	Data control	Data control
Single point of failure	No	Yes	No
Advantages	Implementation is very easily, local measurement and regulation.	Global information and proper co-ordination.	Improved immunity to single point of failure.
Disadvantages	Lack of global information.	Single point of failure.	Complex interaction network.

**Table 2:** Advantages and Limitations of primary droop control.

Types of Control	Advantages	Limitation
Droop Control [64, 65, 66]	Improved power distribution performance.	Ineffectiveness under dynamic situations.
		The simplest scalable low-level voltage/current controller.
	Voltage control and current sharing have trade-offs.	Additional sensors and communication connections are required.
Conventional droop [67, 68]	Avoid critical communication link	Voltage control issues and inefficient power sharing.
Inverse droop [70]	There is no requirement for a centralised controller.	It is power-sharing and not stability.
Non-linear droop [71, 72]	It solves the trade-off and spreads the load.	Improved voltage control under low loads only, not under heavy loads.
Dead band droop [73, 74]	Avoids charging and draining the battery unnecessarily.	The majority of converters in the DC microgrid are regarded to be insufficient for their installation.
Adaptive droop [69]	There are no communication needs, the voltage and current control are substantially better, and installation is simple.	It is inversely proportional to the nth order of SOC.

**Table 3:** Advantages and Limitations of Secondary control.

Types of Control	Advantages	Limitation
Centralized control [80, 81, 82, 83]	Connection of communication with a high rate of speed.	The single point of failure causes it to become less reliable.
		Digital communication link (DCL) is required.
Decentralized control [84, 85, 86]	Less complex and more reliable.	Digital communication link (DCL) is required.
Co-operative control [96, 97, 98, 99]	Regulate the average bus voltage, more flexible and reliable operation.	Sparse communication network is required.
Current voltage sharing control [90, 91, 92, 93, 94]	Voltage compensation without additional controllers.	Low speed bandwidth communication & average current regulation.
DC bus signaling [95]	useful in power management without communication link	Not scalable due to high cable resistance and voltage drop

**Table 4:** Comparison of three control of Hierarchical Controller.

Primary control [62, 63, 64, 65, 66, 67, 68, 69, 70, 71, 72, 73, 74, 75, 76, 77]	Secondary control [80, 81, 82, 83, 84, 85, 86, 87, 88, 89, 90, 91, 92, 93, 94, 95, 96, 97, 98, 99, 100]	Tertiary control [59, 60, 61]
• Stable supply of DC voltage.	• Optimal utilization of renewable sources and storage capacity.	• Scheduling of sources within microgrid.
• Microgrid voltage with in the specified value.	• Regulate the voltage level.	• Economic dispatch.
• Compensate for instantaneous mismatch between schedule power and demand by load.		• Power and energy management system.
• Preliminary power sharing.	• Reduce the voltage deviation and improve the current sharing accuracy simultaneously.	• Interaction with other microgrid.
• Preventing the circulating current.		• Control the power flow between the microgrid and the utility grid.

DC-Microgrid has the benefits of high performance. It may be more useful than AC microgrids. The system avoids the need for generator synchronisation, reduces the usage of converters, and allows different types of distributed energy resources (DERs) and loads to connect to the microgrid common bus through simpler interfaces. Reduced to that with AC-DC electricity transfer, a more rational interface with various renewable resources, an energy storage system, and flexibility with consumer electronic product demands. So, DC Microgrid is commonly used [6]. It also has a low reactive power supply, high performance, and the removal of the converting cycle. The stages of power conversion in microgrid are fewer [7]. DC Microgrid Island, as well as a load-shedding technique focused on two separate DC voltage ratios, in order to make sure a continuous power supply to some of the most essential loads [8]. Furthermore, as its AC counter parts, DC microgrid would not have problems with reactive power supply, synchronization, and harmonics [9].

**Figure 1:**
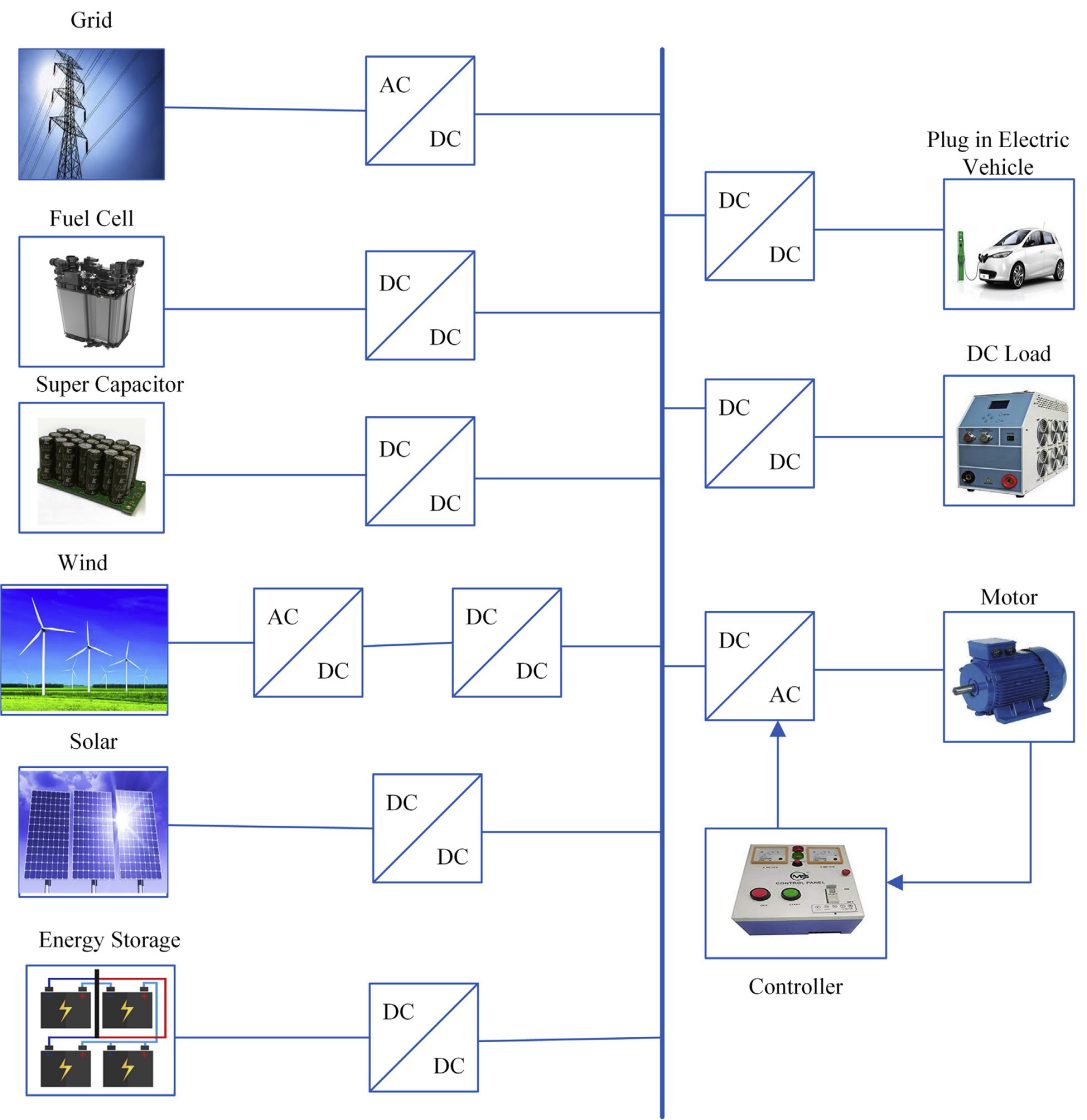
Architecture of the DC microgrid.

Figure 1 illustrates the basic design of a DC Microgrid structure. It consists of several micro sources, energy storage system, energy transfer system, and load control system. The DC microgrid can be run in island mode control otherwise in grid mode control [10]. Furthermore, the DC microgrid is a dynamic multi-target control system that deals with load sharing, voltage restoration, power management problems, exhibiting several time-scale properties. DC microgrid hierarchical control system could be categorized into three systems: a) primary system control b) secondary system control c) tertiary system control [11]. The primary level is controlled by the bus voltage in a microgrid. The difference in voltages that are produced will be reduced at the secondary level, which guarantees smoother operation at the tertiary level. The present flow from/to an external DC source is controlled by the tertiary power [12].

Furthermore, to remove the present sharing deviation between the microgrid modules, a non-linear droop approach including high-order polynomial should be used [13]. Rather than the linear droop solution, to improve the current sharing efficiency impact of the DC microgrid, a non-linear droop with a droop curve slope can be modified. Nevertheless, there might also be a drawback between voltage control and current precision for sharing [14]. In recent times, by coordination between different units in the microgrid, a study of DC Microgrid control, adaptive stability analysis, and stabilization strategies has been introduced, and three major organized control methods are characterized: autonomous, unified, and distributed control. In addition, some major impedance parameters and standards of stability are being reviewed [15].

In general, hierarchical control structures can be categorized into two major types: two-layer control structures and three-layer control structures. Both two-layer and three-layer control schemes of a DC Microgrid were simultaneously tested to increase the control capability in a primary stage. In order to maximize both energy efficiency as well as stability of the system, its strengthened methodology is applied by using the control scheme of the DC bus bar voltage system. Nevertheless, the issue of voltage restoration was resolved in the secondary level system in the microgrid device [16]. DC microgrid clusters help DC microgrids operate more efficiently and provide shared power storage. Establishing DC microgrid clusters by linking neighbouring microgrids is another choice for increasing performance. In this way, in the case of emergencies, each microgrid would be capable of absorbing power from its neighbours. The voltage management and power flow control of DC microgrid clusters are excellent [17].

This review paper is segmented into six segments. In Section 1: Importance of DC microgrid. Section II: Application of DC microgrid. Section III: Market segment in DC microgrid. Section IV: Control topology of microgrid. Section V: Hierarchical Control of microgrid. Section VI: represent the conclusion.

## 2 Application of DC microgrid

**Figure 2:**
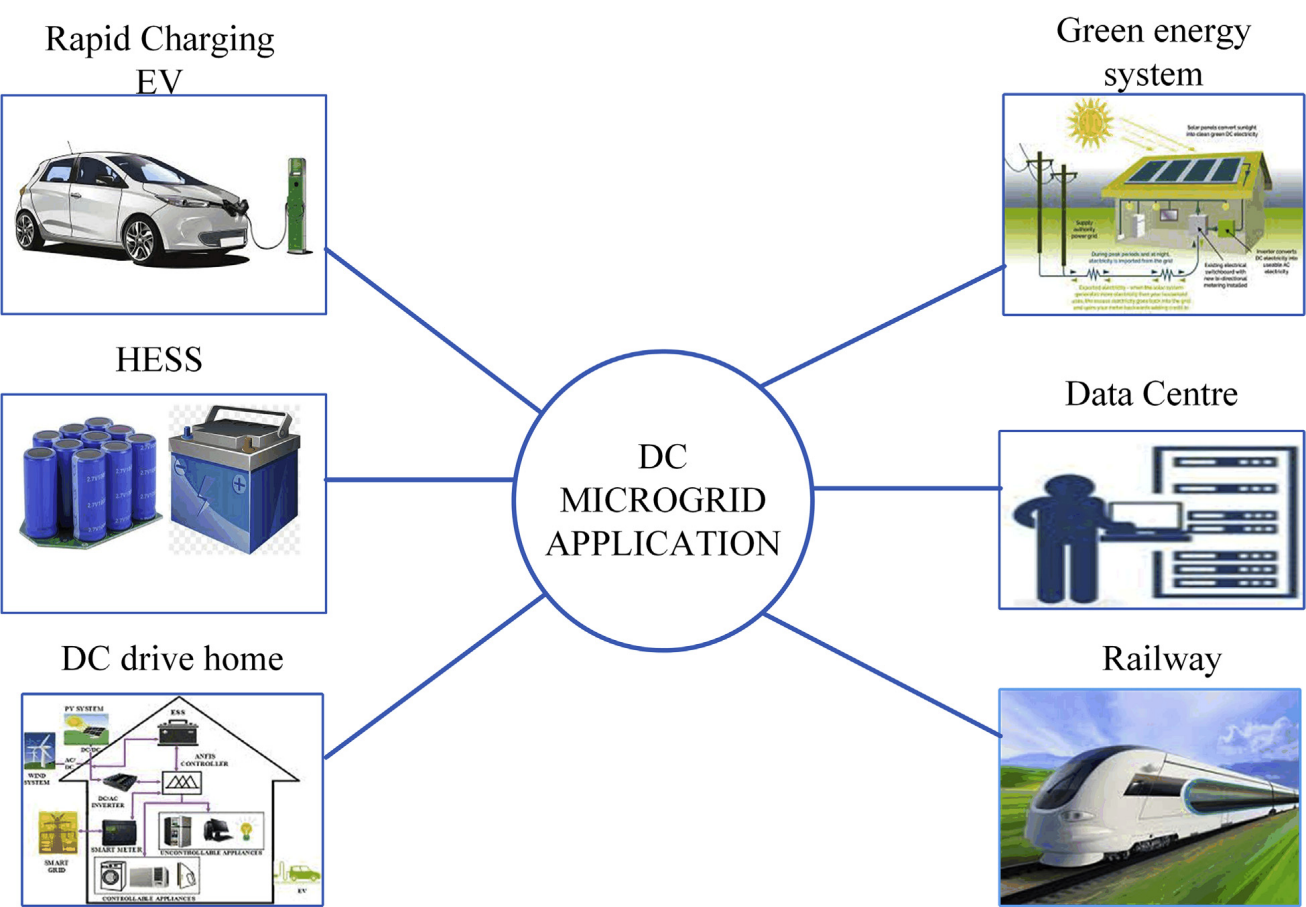
Application of DC microgrid.

Figure 2 illustrates the application of a DC microgrid. It discusses the applications of DC grid systems. DC microgrids are highly recommended for fast-charging electric vehicles, hybrid energy storage systems, DC drive homes, green energy systems, data centres, and railways.

### 2.1 Hybrid energy storage system (HESS)

The hybrid energy storage system (HESS) is a safety device that consists of Bidirectional converters with a half-bridge symmetrical and soft switching connection to a battery and a supercapacitor. It regulates the charging and discharging of the super capacitance (SC) to the battery in an effective manner. When braking and running at a steady pace, the SC energy can be easily replaced, making up for the shortage of the conventional control technique. Controlling the SC's state of charge through the EV's rpm, on the other hand, allows the energy storage device to be more flexible and adaptable [18]. A thorough overview of the design approach for HESS is presented, as well as possible modules for extending the life of batteries in stand-alone photovoltaic power systems.

Solar photovoltaic power generation systems can be grid-connected, independent, or hybrid. In an independent operating system, the energy storage unit stores the excess solar energy for inadequate or no sunlight. The energy storage system composed of battery packs can smooth electric energy fluctuations caused by solar light intensity and compensate for voltage dips or sudden rises in the grid system. However, due to its limited number of charges and discharges, high current charge and discharge times are slow. Using capacitor banks in solar PV systems will make grid-connected electricity generation more feasible [19]. With coupling batteries and supercapacitors together, it reduces the current stress on the batteries in order to decrease its size, improve its lifetime, decrease the depth of discharge of the battery and ultimately reduce the operating and maintenance cost of the system [20].

### 2.2 Rapid charging of electric vehicles

Traditional and advanced control electronic architectures with modern technology allow for both the usual and performance of quick conductive charging. For the next generation of automobiles charging stages are available. The projected challenges with hybrid and electric charging technologies with a focus on existing and potential future advancements for both fixed and active wireless charging systems [21].

The electric vehicle (EV) model has gained a significant amount of recognition as a possible replacement for existing automobiles because of its high ability to produce common vehicles for substantial implementation. The implementation and availability of quick charging architectures, which allow battery replenishment in less time, is a crucial consideration in plug-in electric vehicle (PEV) penetration. This charging station model can support the DC microgrid in various ways, including maximum power saving, variable frequency, reactive reimbursement, and energy variability avoidance [22].

### 2.3 DC driven homes

To ensure appropriate energy capability and to permit the usage of ac and dc applications in an off-grid state, distributed generators can be placed into dc homes via power converters [23]. Hybrid fuel cell/supercapacitor power sources, a roof – mounted photo voltaic panel, and house appliances optimised for both ac/dc applications and an electric charging point are all part of the smart DC home under investigation [24].

### 2.4 Data centres

Data centres are likely to demand greater and more effective electric power, and often a solution to eliminating DC microgrid electric losses. DC microgrids have been investigated with the intention of much more effectively serving modern loads and combining distributed energy storage through the use of data centers. Data centers provide application support and management for a wide range of data processing applications, including information security, telecommunications, internet, intranets, and web hosting [25].

### 2.5 Green energy systems

The country seems to become entirely sustainable when households employ energy-efficient DC-operated DC instruments and generate power from the photovoltaic (PV) modules on roof-mounted buildings [26]. A DC microgrid operated by a rooftop solar can be linked to the grid with an ac-dc converter everywhere the grid is available, integrating a small battery and operating dc-powered dc appliances are identified [27].

### 2.6 Railways

Train transportation is one of the most valuable ways to meet mobility demand due to the low power usage to transportation capability ratio. Rail transportation is perhaps the most viable approach for addressing transportation requirements in terms of its low power consumption to transportation performance proportion, and ecological quality than other forms of transport [28]. Various methods for increasing the reliability of new high-speed trains, the electric traction system allows a railway vehicle to mix conventional braking with an electronic braking system. The train's kinetic energy is transformed into electrical energy. Regenerative braking provides electrical power that can often be collected on commission or transferred to both an overhead transmission line. The impact of regenerative braking and the use of a battery system in a DC rail system [29]. Additionally, the problems in reactive power flow or rather frequency adjustment whenever modules are coupled along the DC bus does not exist [[30], [31]].

## 3 Microgrid market segment

**Figure 3:**
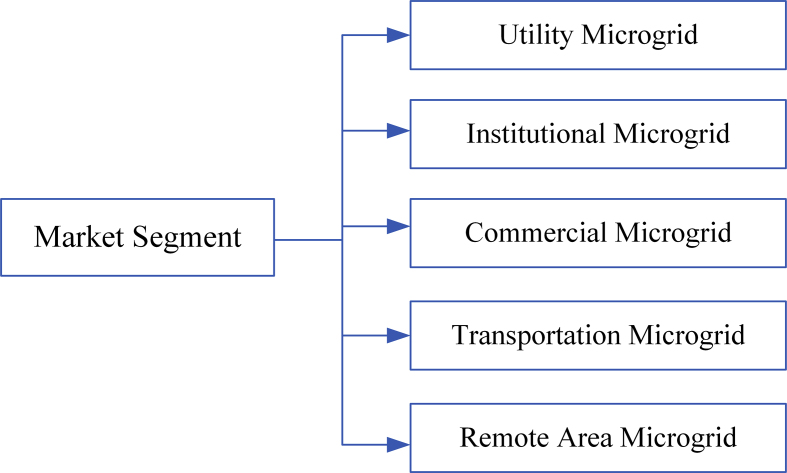
Market segment of microgrid.

The classification of market segment as illustrated in Figure 3 can be categorized into utility, institutional, commercial, transportation, and remote area microgrid.

To solve the aforementioned issue and improve microgrid-integrated delivery system performance, a market-based microgrid Model for optimum scheduling is proposed. In cooperation with the distribution market regulator, a microgrid was operated for the power supply industry [32]. To facilitate the development of a highly efficient way at the distribution system level to exchange electricity and power system resources, In the commercial energy market, a Distributor Market Operator (DMO) and an Independent System Operator (ISO) might collaborate. Instead of one for each utility, an independent DMO might provide a universal market environment [33].

### 3.1 Utility microgrid

The utility correctly predicted a load of microgrids in its area of support and collected everything to the distribution centre utilising existing processes [34]. The utility sends the individual costs to microgrids until the energy price is decided in the wholesale market [35].

### 3.2 Institutional microgrid

An Indian corporate energy infrastructure was studied for technologic economic efficiency analysis as an intelligent micro-grid within the market energy pricing dynamics. A smart micro-grid based on an Indian institutional electricity infrastructure was evaluated for technical and economic performance under market energy price conditions [36].

Institutional infrastructure a large number of potential for creating a PV-based microgrid to reduce their grid peak demand. In both grid-connected and islanding modes, a PV-based power system with an energy storage battery and distributed generators have a lot of potentials to act as a microgrid [37]. On the weekdays, the average institutional load absorbs more resources than on weekends. The active involvement of battery storage in the institutional microgrid, as well as its collaboration with other DERs, will improve the institutional energy system's stability, security, and cost-effectiveness [38].

### 3.3 Commercial microgrid

Shopping malls and manufacturing parks, as well as the mining, steel, fertilizer, and oil and gas sectors in all use commercial microgrids only [39]. The continuous need for an uninterrupted electricity supply to carry out smooth industrial processes in order to reduce downtime, increase productivity levels, and reduce equipment damages is responsible for the segment's development. The increased adoption of microgrids for commercial use has resulted from the requirement for commercial and industrial sectors to meet carbon reduction targets [40].

To control resources in commercial microgrids, a hybrid approach based on demand management and multiple agent system is used. Demand-side management is important because it regulates business operations by reducing energy requirements at peak consuming hours. The multiple-agent system was considered a form of knowledge based on computation, that is being used to lower global market costs and total loads. Using a hybrid approach based on optimizing production rate, energy demand is reduced while costs are reduced by a percentage [41].

### 3.4 Transportation microgrid

Transportation is the most important foundation of global economic prosperity. Transportation is mostly a product of fossil fuel, which has a significant environmental effect. The electric vehicle transportation environment is a fundamental field that needs special attention, since it is one of several areas where microgrids can make a significant difference [42].

The existing electric vehicle charging system is as "dirty" as the local power grid, and new technology technologies, such as solar energy, energy storage, and microgrids, will allow a more efficient, universal, and smarter electric vehicle charging infrastructure if they are integrated. Microgrids' efficient electric vehicle charging system should be something that needs to be widely available and accessible [43]. Electric charging stations would need to be established in three kinds of regions: on highways, along roads, and at public transit hubs like airlines, buses, and rail stations, in addition to office buildings and urban areas [44].

### 3.5 Remote area microgrid

DC microgrids are becoming more common as a more powerful and easy power system, especially in remote areas where the main grid has yet to be installed. It is also known as a stand-alone microgrid. It is a possible solution to the issue of rural and remote areas without electricity. In a stand-alone microgrid, wind turbines and photovoltaic panels will coexist or coexist only partially. For power balancing and system stabilization, the system is a stand-alone microgrid that uses an energy storage system as the primary power source [45].

These hybrid energy storage methods involve centralised management to meet a stand-alone microgrid's overall consumption need. Connecting overloaded remote area microgrids to neighbouring microgrids helps alleviate load-shedding [46]. Power electronic converters have made it possible to use DC loads and DC converters in several circumstances [47]. In addition, DC-based distributed energy resources and diverse energy storage devices demonstrate a major opportunity for DC microgrids [48].

## 4 Control topology

**Figure 4:**
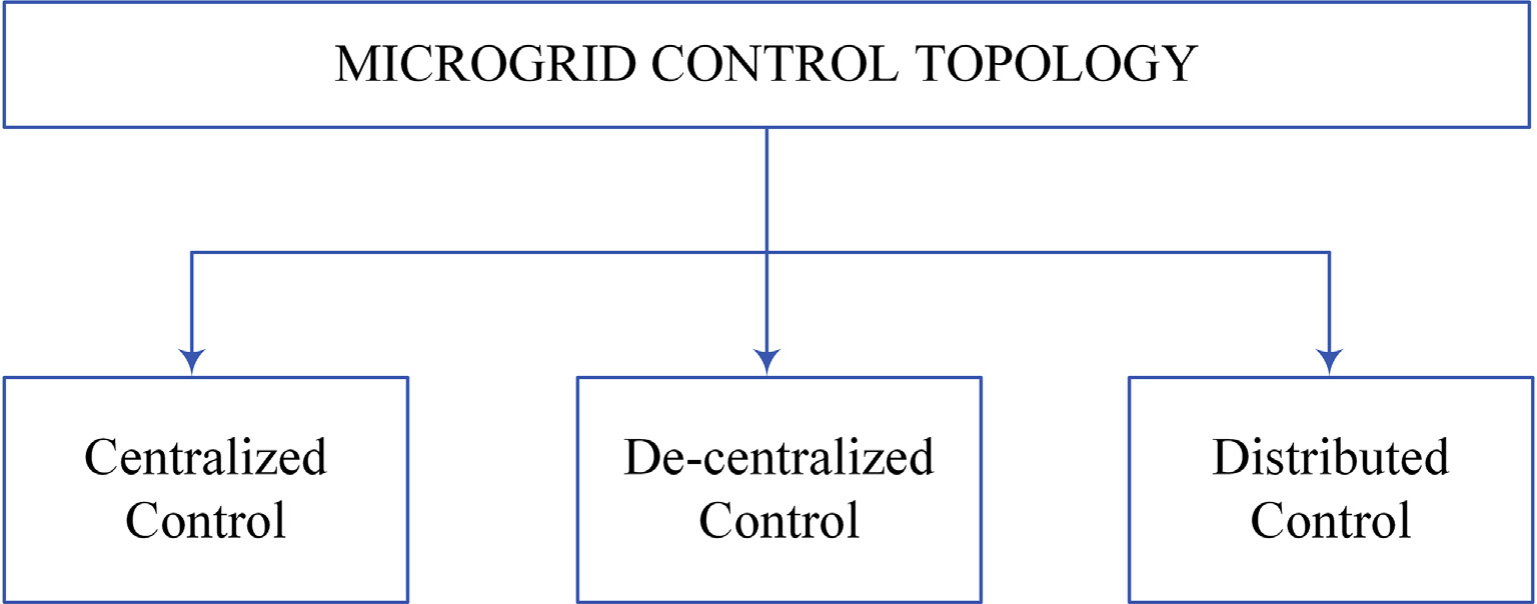
Control topology of microgrid.

The control topology of the DC microgrid is illustrated in Figure 4. For the stable activity of the DC microgrid various control aspects are used such as Centralized control, Decentralized control, and the last one is the distributed control aspects [49]. Table 1 shows the benefits and drawbacks of decentralised, centralised, and distribution control.

### 4.1 Decentralized control

**Figure 5:**
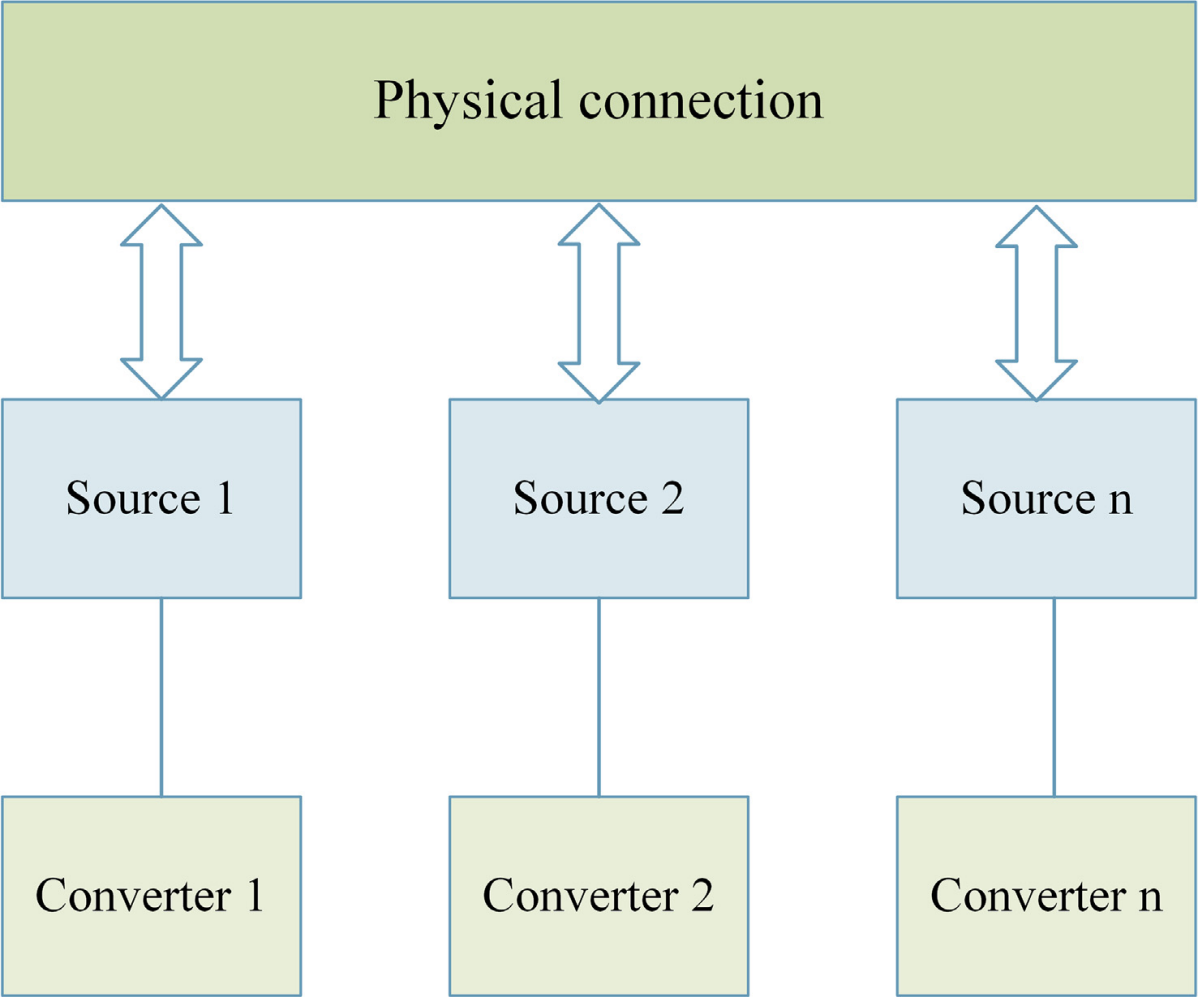
Decentralized control.

Decentralized control can be implemented in a source-based DC microgrid shown in Figure 5. There is no single owner in a decentralized society. Instead, they depend on several central owners, several of whom keep a copy of the services that consumers have access towards. In a DC microgrid, there are decentralized approaches that can manage the output of multiple parallel converters [50].

The analysis of DC bus voltage differences, primarily in the common DC bus voltage, enables coordinated operation across diverse distributed units in a DC microgrid. A decentralised control technique has been presented to achieve the economic performance of a DC microgrid in both grid-linked and islanding modes. A cost-effective power-sharing method among several types of distributed generators was developed without the usage of a microgrid centralised controller, effectively reducing the daily electricity bill [51].

### 4.2 Distributed control

**Figure 6:**
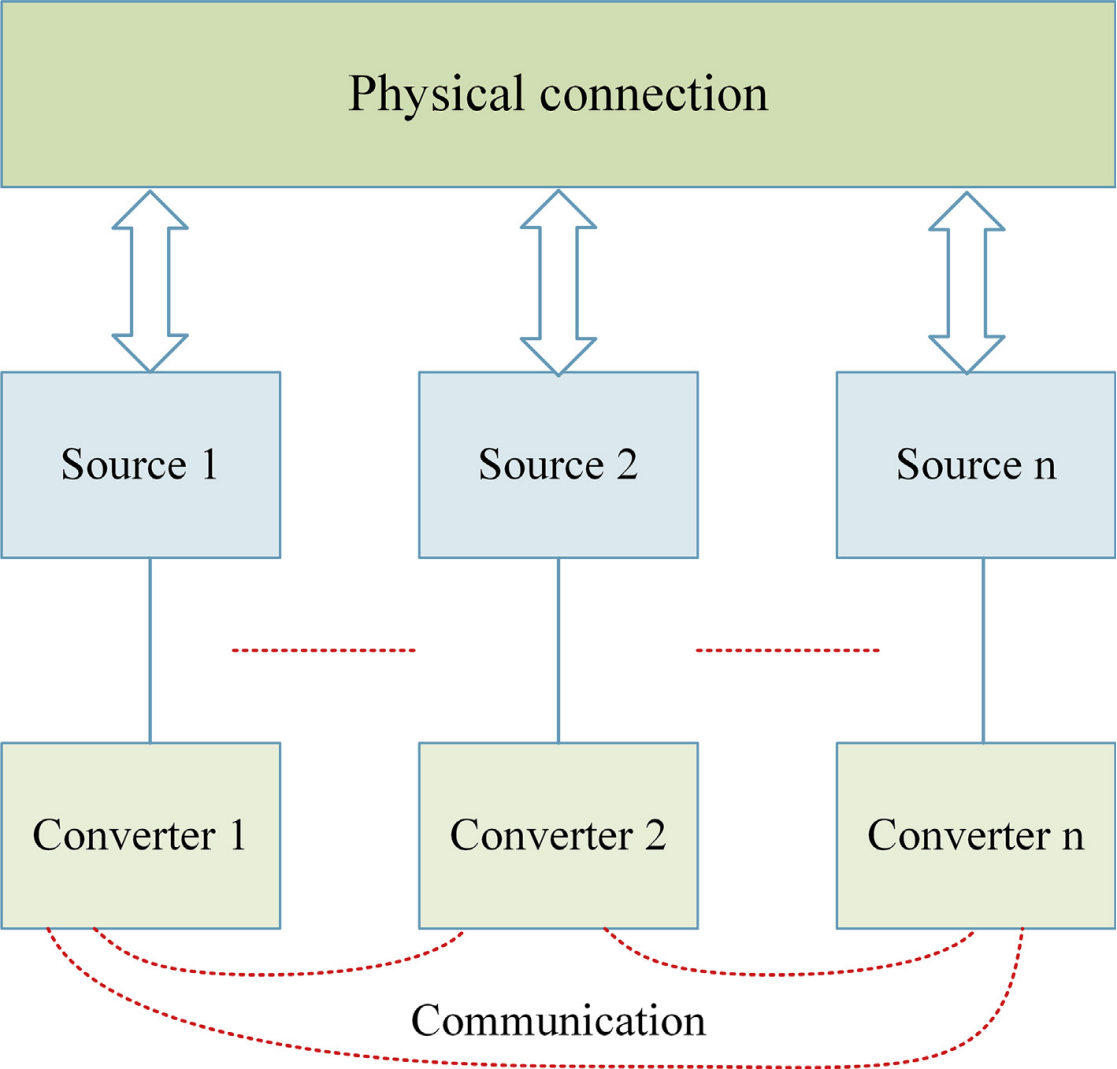
Distributed control.

Distributed control can be implemented in a source-based dc microgrid with communication shown in Figure 6. The distributed system allows sharing data ownership. Users are often given access to hardware and software resources to increase the system's performance in certain situations. A distributed system is protected from element failures that occur independently, which would significantly increase its system performance. The communication line connects every distributed unit to all the others [52].

Finally, it is easy to conclude that distributed control, for its many benefits, can provide even more functionality than decentralized control. It indicates a lack of difficulty analysis of result and unsuitable settings, that was characterized with communication time delays and sensor measurement errors. Bus voltage deviation, power monitoring error, and analytical performance complexity are the primary disadvantages of distributed control schemes [53].

### 4.3 Centralized control

**Figure 7:**
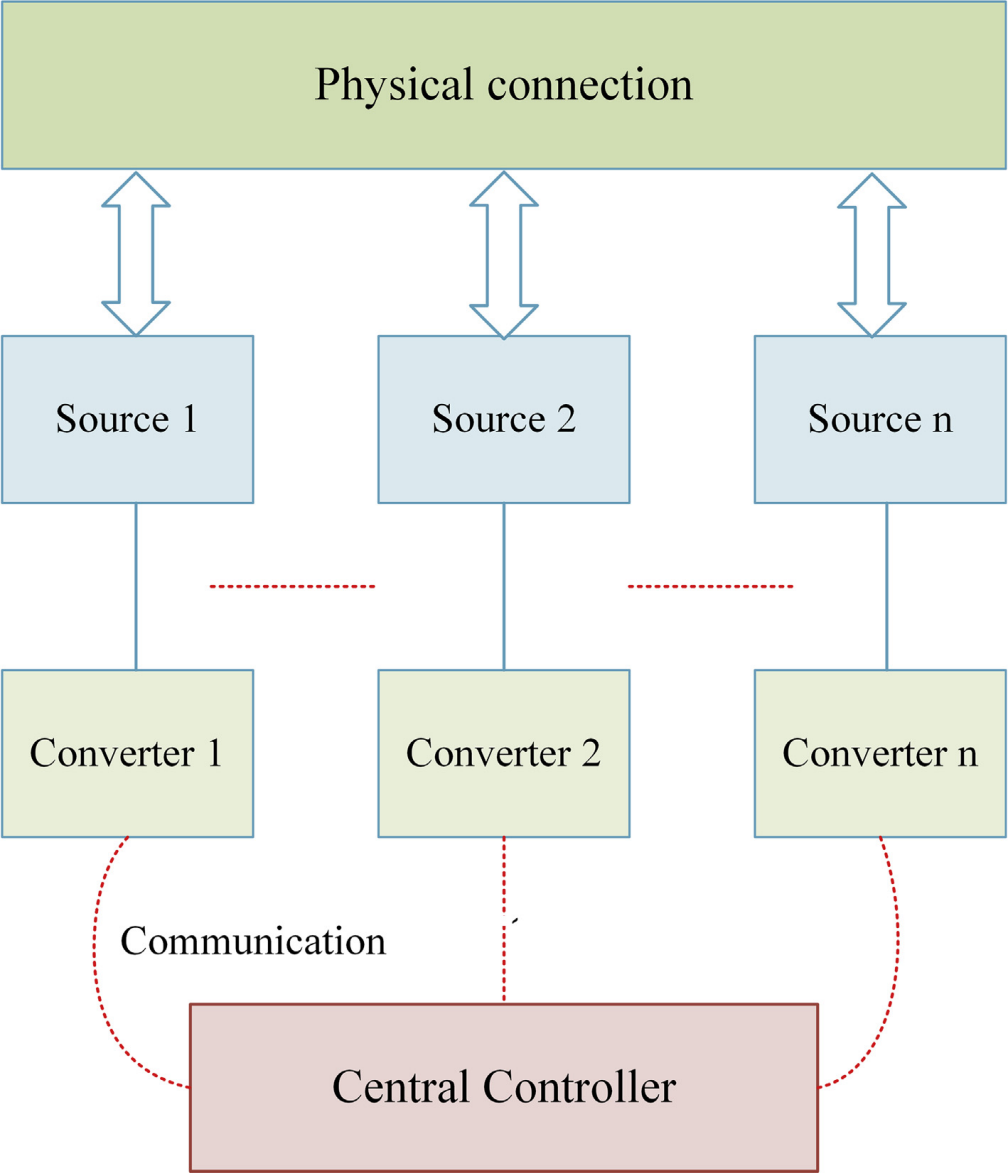
Centralized control.

Centralized control can be implemented in a source-based DC microgrid shown in Figure 7. Both users are linked to a central network owner or "host" in a centralized environment. The central owner keeps data and user information that all people can use. User accounts, user-generated content, and other types of information may be included in this data. A centralized structure is simple to understand and can be easily developed. It's useful to classify it as a DC microgrid, which connects sources and loads via a centralized controller and computerized communication network. Because it establishes a scope between multiple control levels, hierarchical control is also a safer alternative for a big DC microgrid [54].

Furthermore, hierarchical type control is more accurate than centralized control since it stays active even when centralized control fails [55]. Control aspects are used to solve the following issues in the DC microgrid: maintenance of DC bus voltage, power quality, and load sharing. Hierarchical control is implied to tackle these problems and provides various control aspects even in the event of centralized control failure. It continues to be operational [56].

## 5 Hierarchical control

**Figure 8:**
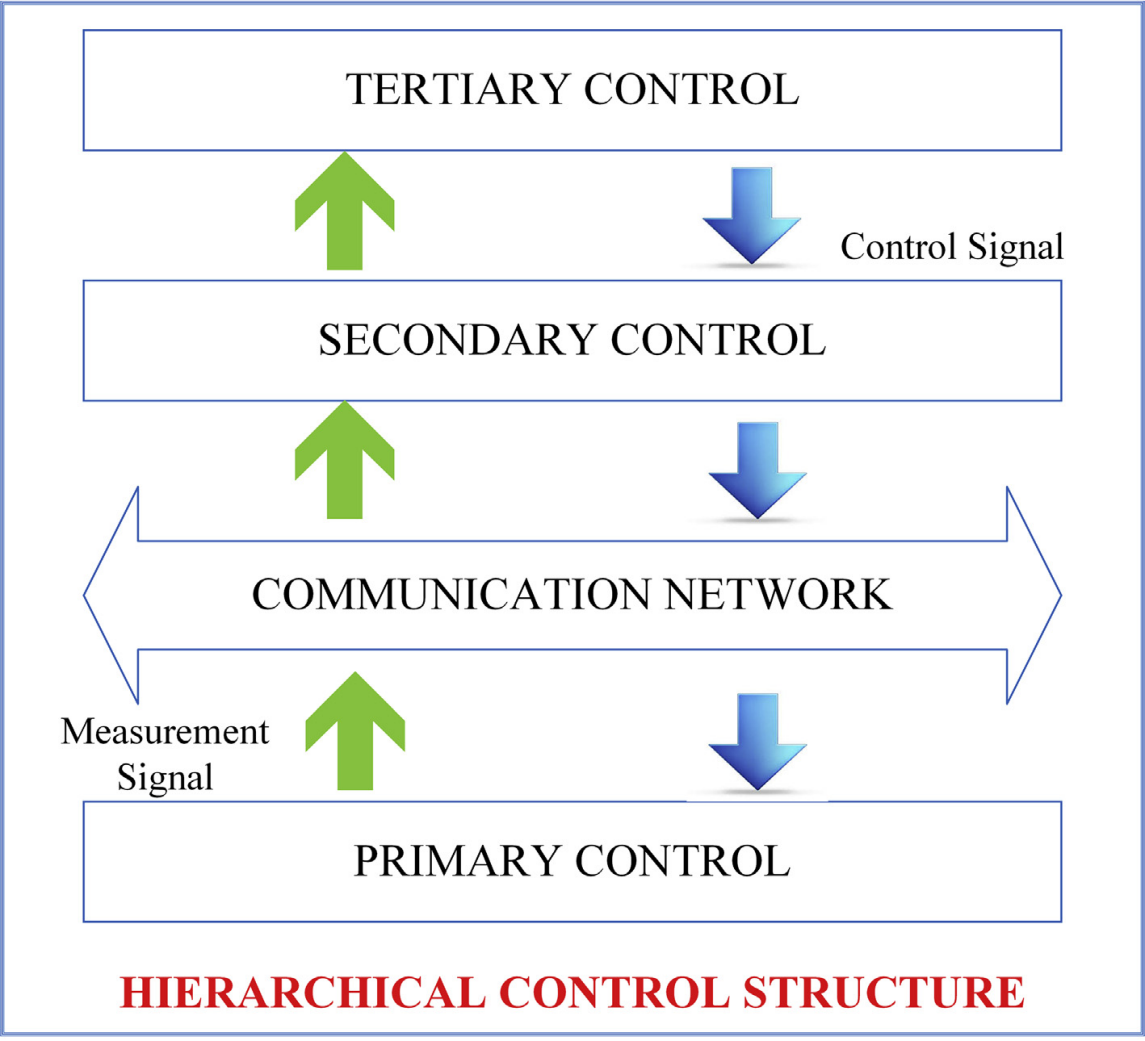
Architecture of hierarchical controller.

**Figure 9:**
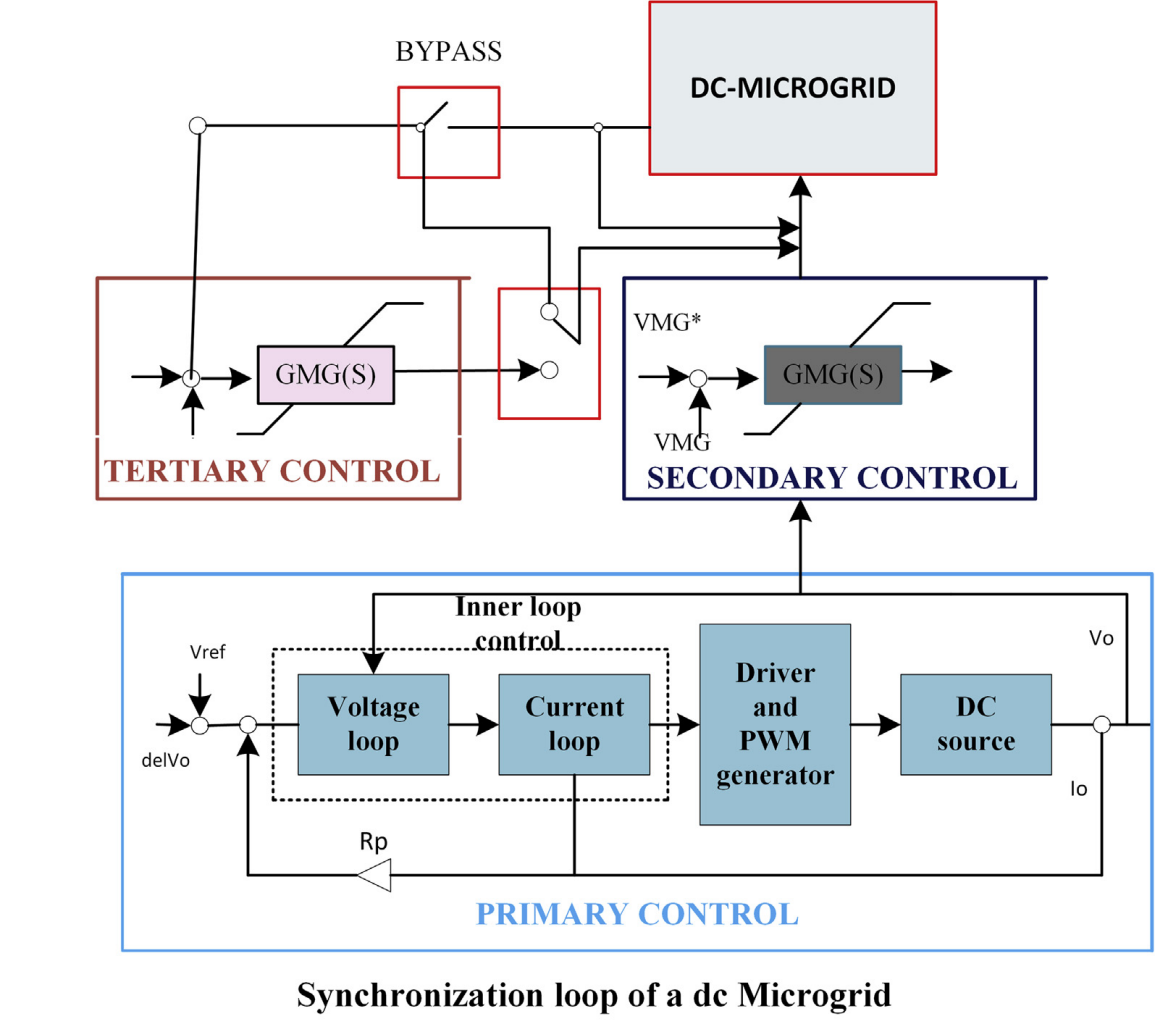
Schematic diagram of Hierarchical controls of a DC microgrid.

The structure of hierarchical control was illustrated in Figure 8 and schematic diagram of hierarchical controls of a DC microgrid was illustrated in Figure 9. In the architecture is categorized as Tertiary, Secondary and Primary control [57]. Electrical grid is in charge of current and voltage management. Secondary control, to a greater extent than primary control, interacts with voltage compensation and increases current sharing [58]. Table 4 shows the benefits and drawbacks of hierarchical controller.

### 5.1 Tertiary control

In hierarchical control topology, the primary control does not have any communication between the systems. The current will be shared between the microgrid systems and also regulate the voltage in the DC bus. If there is any deviation in voltage, it will be restored by secondary control by using droop control of lower bandwidth communication. If there is a large voltage deviation, controlling power flow is impossible in centralized controller. To solve the issues, decentralized controller is used [59]. A higher level of control strategy in tertiary which includes optimal operation and economic dispatch in microgrid and acts as a master controller that collects the generation data and loads data via supervisory control [60]. In grid-connected mode, it will monitor and control the utility of the microgrid to minimize the running cost [61].

### 5.2 Primary control

**Figure 10:**
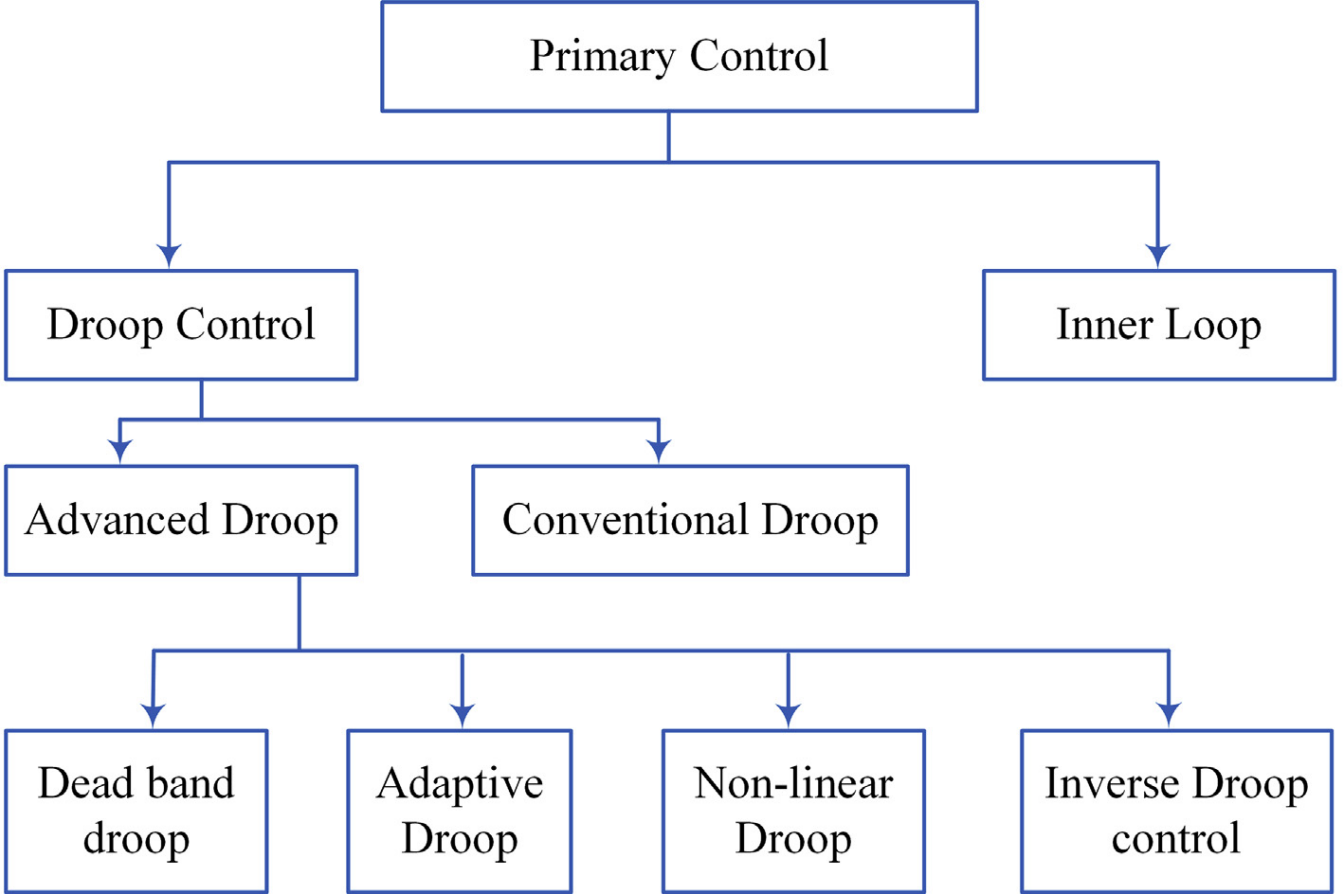
Topology of primary control.

The topology of primary control is illustrated in Figure 10. To interface between the sources and loads, the DC microgrid is an important tool that can be controlled. The inner loop and droop controls are the controllers of the primary. The inner loop control regulates the current and voltage [62]. The droop control includes conventional droop controls that will inherently trade-off in a microgrid. Local controls for the different sources are configured using the droop method to ensure that the sources are used efficiently and reliably. A low-bandwidth networking system links the local controllers to the control center. Finally, adaptive droop control methods are introduced [63]. Table 2 shows the benefits and drawbacks of primary droop control.

#### 5.2.1 Droop control

Droop control is commonly used for DC microgrid due to its natural flexibility. The droop control approach is widely used to adjust amplitude and power sharing in microgrid DC buses. The voltage and current droop are segmented into droop control strategies in the dc microgrid. A comparative analysis of current and voltage droop control solution with an emphasis on state of equilibrium power-sharing efficiency and consistency was performed [64].

In the current droop of the DC bus voltage, reference current is generated, while in voltage droop in load, the reference voltage is produced. The appropriate power supply-based voltage droop control approach is used for effective control. The output power can be controlled by droop control techniques with improved efficiency and power sharing is achieved [65]. Droop control enables a photovoltaic unit to the betterment and manages output power using a basic control scheme that does not necessitate a complete power measurement. Not only energy saving as well as load shedding and renewable energy supply control are critical in an islanding power grid with a diverse spectrum of renewable energy sources. Droop control for DC systems is more reliable while compared to other control strategies because there is no failure point, and it only requires bus voltage details [66].

#### 5.2.2 Conventional droop control

The potential and current/power and potential are the control strategy of the basic droop control. The microgrid-based power converter voltage is controlled with control methods of droop characteristics. The power-sharing and stability of droop control schemes for current mode and potential mode in voltage source control-based dc microgrid were contrasted in this analysis. A DC current controller is used in current-mode droop control to manipulate the injecting current on every terminal depending on the measurement of voltage. The terminal voltage is controlled by the potential mode droop power, which is based on current measurements [67]. If the output current grows in this sort of droop control for the dc microgrid, the voltage relation is linearly reduced, resulting in the power-sharing process. Despite the fact that droop control is often employed as a decentralised technique for load power sharing, there are a few things to keep in mind. Average voltage and average current Proportional Integral (PI) controllers are used to improve load current sharing accuracy and recover the local DC output voltage [68].

#### 5.2.3 Advanced droop control

To increase the system efficiency various droop characteristics are analysed. The load regulation and good trade-off in inverse droop control topology. The output voltage can be raised with a suggested inverse droop regulation [69].

#### 5.2.4 Inverse droop control

Decentralized control for an input sequence output parallels modular device can be gained by using the inverse-droop approach. Power sharing can be done effectively. The power-sharing achieved in this control can be performed in both steady state and transient analysis, with sharing of output and input current. Furthermore, the performance control feature can be enhanced. Since the input voltage has no effect on the output voltage link [70].

#### 5.2.5 Non-linear droop control

Non-linear droop topology was proposed for dc systems to increase load sharing and voltage management. Instead of employing continuous droop resistance, this is recommended. The output current determines the droop resistance value. The droop resistance increases when the load is raised. It achieves increased load sharing under heavy loads and improved voltage balance under light loads. It solves the trade-off in the traditional droop approach [71].

The non-linear droop control solutions that have been developed have greatly reduced the trade-off between voltage management and load sharing. To improve the present accuracy of sharing and to increase the overall system stability, three unique non-linear droop control techniques were suggested, processed, and developed. Match the droop gains of several droop curves with a higher-order polynomial droop curve to boost load sharing even more. The best voltage regulation is provided by implementing the polynomial droop gain techniques. In all loading conditions, the polynomial droop curve method provides effective load sharing and considerable voltage control [72].

#### 5.2.6 Dead band droop control

Due to the massive invention, a lithium-ion rechargeable battery uses the dead-band droop characteristic, which requires the battery to run in "offline" mode. A floating or standby mode of operation is used to avoid the frequent charge and discharge timing [73]. Droop control modifies the DC injection current to its reference value, allowing control mechanisms for battery-based energy storage systems to be built. An adaptive droop characteristic for the energy storage system improves device stability by minimizing dead band errors and reducing unnecessary switching disturbances [74].

#### 5.2.7 Adaptive droop control

An adaptive droop system is used in the primary control layer, allowing local controllers to operate autonomously and flexibly in the face of disturbances such as faults, load fluctuations, and environmental changes. For optimum power, in the primary control layer, an adaptive droop technique is required, which allows local controllers to move effectively and dynamically over the presence of disturbances including breakdowns, load oscillations, and ecological factors. A higher control level adjusts voltage reference points depending on customised energy optimization decisions for optimal power distribution. It's being used to control voltage and ensure proper load distribution. A higher control layer adjusts voltage points of reference based on tailored energy optimization choices. It is used to regulate the voltage and achieves good load sharing. If a different source of power is taken into this control, then it will not be accountable. The excellent control of voltage and power sharing is achieved by changing the constant droop rather than changing the smaller or larger droop control [75]. In the analog circuit, the current sharing output is increased, which essentially improves the droop gain. The greater voltage droop is balanced by raising the voltage at a certain interval due to a rise in droop gain. Droop gain results in effective voltage control and correct current sharing. Droop gains dynamically adjust depending on the loading state [76].

#### 5.2.8 Inner loop

Inner loop deals with AC/DC converters and DC/DC converters. The AC/DC converter is made up of a current controller that is established inside the loop. The current controller generates the optimal pulse width modulated switching signals because it supplies the output current nearest to the reference and has the best convergence rate to the point of equilibrium where the power factor is unity. In other words, the current controller provides the best potential additional functionality [77]. Although there are many different kinds of DC/DC converters, their controls may be split into two categories voltage control mode and current control mode. In voltage control mode, the DC/DC converter acts as an adjustable voltage source in addition to setting the voltage reference. When operating in current/power control mode, the converter acts similarly to a controlled current and power source [78].

### 5.3 Secondary control

**Figure 11:**
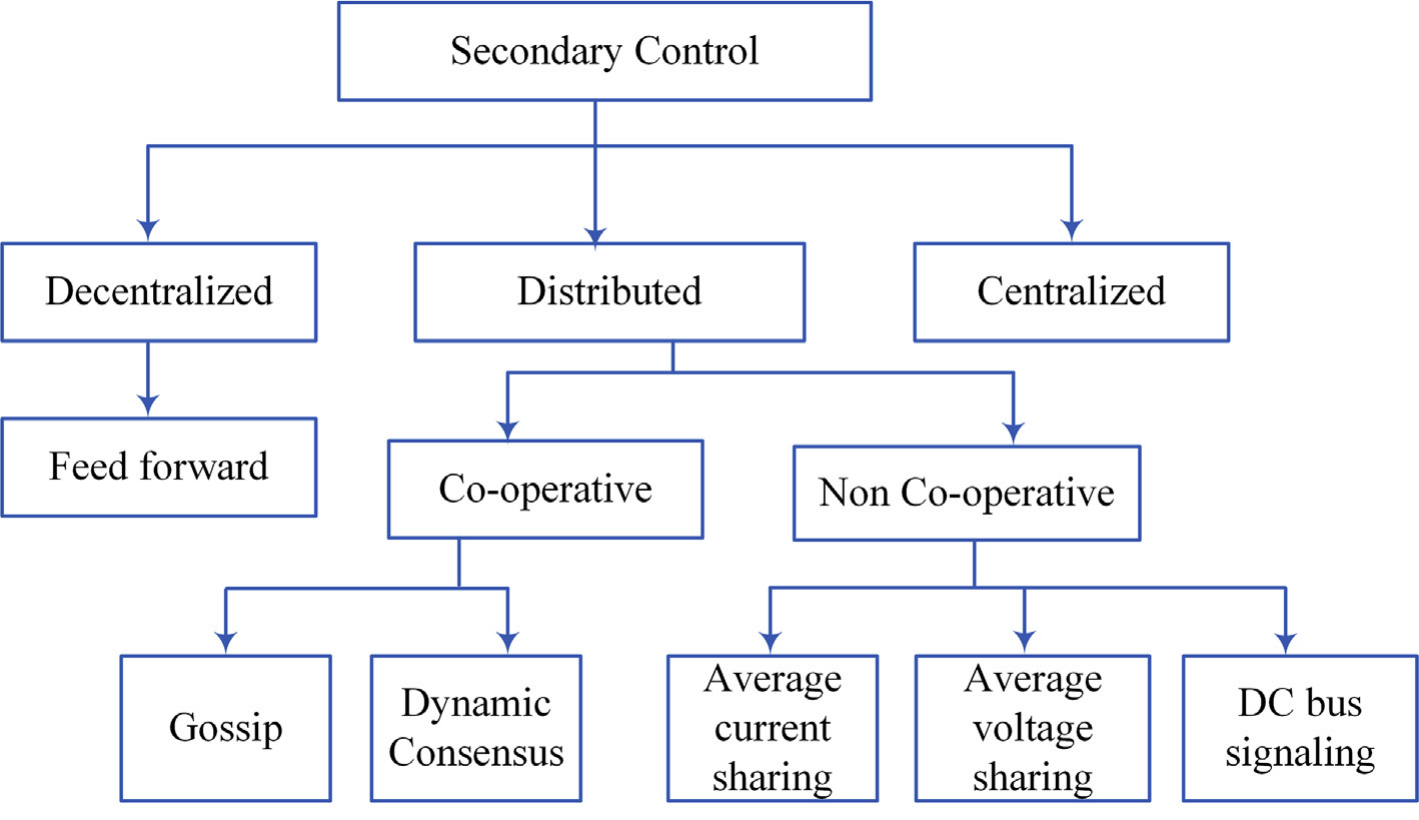
Topology of secondary control.

The centralized control distributed control, and decentralized control are the three control strategies of secondary in DC microgrid as illustrated in Figure 11. As one can see for centralized and distributed control, a digital communication connection is needed. This will decrease both control strategies reliability, especially for centralized controllers the voltage failure exists. Distributed control strategies have been recently stimulated such as sharing of current. DC bus signaling techniques are summarized to work out the entire network information [79]. Table 3 shows the benefits and drawbacks of secondary control.

#### 5.3.1 Centralized controller

The input sources and loads are connected with the network of digital communication by implementing a centralized controller based on a DC microgrid. Through network communication, the centralized controller sends a signal to every module to adjust the voltage [80]. The multi-layer supervising component is used for Power leveling, and energy managing in the photo voltaic penetrated microgrid. A conventional three-level hierarchical control system, sharing of loads and managing of voltages are suggested [81]. The voltage variation caused by voltage droop is removed using a PI controller for secondary regulation [82]. For Island DC microgrid, the reference provides a DC bus signaling secondary control [83].

#### 5.3.2 Decentralized control

In order to control local regulation, decentralized control uses local measurements. If it is not necessary for communication between sources, centralized controller is additionally made to enhance the device's efficiency [84]. The proportional controller will correct the impedance effect in secondary control. Locally distributed control devices are calculated and transmitted. For proper power sharing line impedance is canceled by using approximate gain. Furthermore, the reference voltage in every module is added in a forward feed term. The bus voltage and load current often following a characteristics of droop no extra voltage controller needed to calculate the load current [85]. Multiple loads are connected and supplied to the microgrid. The main feeder load current is measured and supplies all loads with electricity [86].

#### 5.3.3 Distributed secondary control

Even though there is no centralised control device, distributed techniques are indicated. The major advantages of this strategies are that even if some communication links fail, the device will retain full functionality [87]. Unlike centralized control, only available variables are present in the information directly shared between local controllers [88]. In the absence of a direct communication connection between the two units. It is limited to directly access the data monitoring. To overcome this problem the signaling of DC bus, sharing of current and voltages are used in co-operative strategy [89].

#### 5.3.4 Current – voltage sharing

The PI controller with improved droop characteristics is used to regulate the current and voltage. The error of line resistance by sharing of the load is eliminated in large droop gain with the increase of the load each droop line is relocated by the same amount [90]. It is important to calculate the average current and identify the shift gain, which is not easily developed and implemented using three PI controllers as a secondary control tool [91]. Utilising distributed control techniques, the overall soc of kilowatt - hours in the batteries is computed [92].

The droop gain is adjusted by the average current controller. In order to compensate for common load, the voltage will decrease and single compensation process is recommended [93]. A highly droop gain is set by the secondary control system for effective sharing of power. In distributed secondary control an average voltage sharing control is suggested by using lower bandwidth communicating channel to its primary controller with a measured average control signal [94].

#### 5.3.5 DC bus signaling

The strategy of distributed control signaling of DC bus is used for energy storage by photo voltaic generation system. The mode switching is determined by voltage bus signaling as an information carrier. The regulation of voltage in DC bus is regulated by modular DC converters to smooth switching between the operation of voltage constant and tracking of maximum peak power [95].

#### 5.3.6 Co-operative control

The co-operative control mechanism is used to adjust the set point of voltage. In co-operative control mechanism, the current and voltage regulator is replaced by the mechanism of loop droop to adjust the set point of local voltage and current for sharing of loads [96]. The control strategies of decentralized are required for voltage regulation operation of DC microgrid bus without bus signaling for balancing of storage of energy and distributed controller [97].

The sharing of current is improved by negative current sharing in distributed control of microgrid-based control of hierarchal [98]. The information is shared by consensus algorithm is used to control the multiple distributed generation in a microgrid. Distributed method of control by consensus algorithm is applied for sharing information to co-ordinate the distributed generation in a microgrid [99]. The quality and stability of voltage and power is regulated in DC microgrid by using DC electric spring [100]. The sharing of power is shared between batteries and ultra-capacitor is distributed in DC microgrid by multi co-operative control strategy without central controller [101]. The repeated failure in the converter and communication network co-operative scheme of control is used. The major advantages are voltage, current, and heating stresses is reduced in the device [102].

## 6 Challenges and future work

In today's energy situation and considering future energy trends, hierarchical control enables DC microgrids to supply energy efficiently and inexpensively. Recently, DC microgrids have had several technical advantages over ac microgrids. For example, harmonics are easier to deal with, adding renewable energy sources, no frequency, and reactive power control issues, and consumer loads can be connected directly to the DC bus. Despite this, there are a lot of technical problems, a challenging controller, and sophisticated operation and planning. DC microgrids need careful planning and operation from system planners and operators to ensure their desired levels of flexibility, dependability, and stability are fulfilled. Multiple sophisticated control strategies may be redesigned/improved to ensure microgrids have concurrent power transfer and accurate DC voltage bus management. The aforementioned control techniques make the microgrid efficient, dependable, and secure in accordance with customer needs; they may also be widely implemented in a smart grid in the future.

## 7 Conclusion

In this review paper conclude that the basic inner loop controller topology of primary Voltage control and balancing performance are indeed poor in load condition. The secondary control technique improves power quality and distributes power more evenly and the communication network is flexible it is furtherly categorized into centralized distributed and decentralized controller topology. Centralized controller is optimized and communicates to all units by a digital communication link. It achieves highly functionality but suffers by a single point of failure. Decentralized controller topology achieves high flexibility and depending on local variables that cause system optimisation problem. distributed controller topology eliminates the single point of failure and achieves higher modularity and reliability. Finally, by concluding this paper, each controller has its own individual characteristics. In recent years for load stability, Hierarchical control strategy been more dependent on DC microgrid. In this case, there is a greater scope of research for DC grids. The depletion of traditional energy resources, the requirement for storage devices, and the growing popularity of renewable energy sources, among other factors, point to the DC microgrid as the foreseeable future grid. The control goal, as well as several tactics, have been considered.

This study discusses power distribution and voltage restoring approaches in hierarchy control DC microgrids. As the principal control, inner control loops targeted at currents and voltage stabilization and initial power-sharing are implemented. The traditional control strategy has poor voltage stabilization at heavy loads and low energy sharing efficiency at part loads. To tackle this, several droop characteristics such as adaptive, inversed, non-linear, and dead band droop are described. A secondary controller connects with all other units via dedicated centralized control schemes. In the DC microgrid a supervisory scheme is used to achieve global optimization or to decide the correct operating modes for each device. For achieving advanced functionalities centralized management has the most flexibility. Decentralized control has a high level of stability and adaptability, which is essentially affected by local factors. It's regarded to be a valuable tool for coping with reference variables including connection issues. The disadvantage is that it does not have a global system. They collect data and organize it by using a cryptographic algorithm.

The major research issues in this area will include control system structure, connection, consistency, and confidentiality. A single controller cannot achieve primary goals including Voltage stability encompasses voltage level, static power optimization, and also power sharing among distribution generators, supplementary services, engagement in electricity market, and operational cost reduction. As a reason, multi-level control is recognized as a conventional control of the DC microgrid under this circumstance.

## Declarations

### Author contribution statement

All authors listed have significantly contributed to the development and the writing of this article.

### Funding statement

This research did not receive any specific grant from funding agencies in the public, commercial, or not-for-profit sectors.

### Data availability statement

No data was used for the research described in the article.

### Declaration of interest's statement

The authors declare the following conflict of interests: The authors whose names are listed immediately below certify that they have NO affiliations with or involvement in any organization or entity with any financial interest (such as honoraria; educational grants; participation in speakers’ bureaus; membership, employment, consultancies, stock ownership, or other equity interest; and expert testimony or patent-licensing arrangements), or non-financial interest (such as personal or professional relationships, affiliations, knowledge or beliefs) in the subject matter or materials discussed in this manuscript.

### Additional information

No additional information is available for this paper.
